# The Distribution of Climate Change Public Opinion in Canada

**DOI:** 10.1371/journal.pone.0159774

**Published:** 2016-08-03

**Authors:** Matto Mildenberger, Peter Howe, Erick Lachapelle, Leah Stokes, Jennifer Marlon, Timothy Gravelle

**Affiliations:** 1 Department of Political Science, University of California Santa Barbara, Santa Barbara, California, United States of America; 2 Department of Environment and Society, Utah State University, Logan, Utah, United States of America; 3 Department of Political Science, Université de Montréal, Montreal, Québec, Canada; 4 School of Forestry and Environmental Studies, Yale University, New Haven, Connecticut, United States of America; 5 Department of Government, University of Essex, Essex, United Kingdom; Stockholms Universitet, SWEDEN

## Abstract

While climate scientists have developed high resolution data sets on the distribution of climate risks, we still lack comparable data on the local distribution of public climate change opinions. This paper provides the first effort to estimate local climate and energy opinion variability outside the United States. Using a multi-level regression and post-stratification (MRP) approach, we estimate opinion in federal electoral districts and provinces. We demonstrate that a majority of the Canadian public consistently believes that climate change is happening. Belief in climate change’s causes varies geographically, with more people attributing it to human activity in urban as opposed to rural areas. Most prominently, we find majority support for carbon cap and trade policy in *every* province and district. By contrast, support for carbon taxation is more heterogeneous. Compared to the distribution of US climate opinions, Canadians believe climate change is happening at higher levels. This new opinion data set will support climate policy analysis and climate policy decision making at national, provincial and local levels.

## Introduction

Climate change poses a pressing global challenge. Yet, success in meeting this challenge will depend on national public policies. In turn, effective and durable greenhouse gas reduction policies require public support. As the ninth largest emitter of greenhouse gases, Canada is one important country that needs to enact ambitious climate policy [1]. Since 1990, oil and gas development from the country’s abundant supply of unconventional oil sands bitumen has driven Canada’s emissions growth, a trend projected to continue to 2020 [2, 3]. These oil sands emissions are significant globally–Alberta’s proven oil reserves represent about one eighth of the total global warming potential from global reserves [4]. Canada is also among the largest fossil fuel producing countries, ranking fifth in crude oil, fifth in natural gas, and twelfth in coal production [5].

At the same time, negative climate change impacts are also disproportionately affecting Canada due to Arctic amplification at high latitudes [6]. Sea ice loss, thawing permafrost, and coastal erosion are already accelerating [7–9], and impacts from extreme weather such as droughts, flooding, and heat waves [10–12], as well as ecological disturbances such as wildfires and pine beetle outbreaks are projected to increase [13, 14]. All of these factors make climate action in Canada both vital and difficult.

Given the significant role fossil-fuel energy plays in the Canadian economy, climate action will be difficult. Implementing a national climate policy would represent a large change in economic and energy policy for Canada. Consequently, Canada is in many ways a difficult case for implementing ambitious carbon pricing. Given geographical variation in energy resources and electricity sources, policy costs and benefits will be unevenly spread across the country. Further, as a highly decentralized federal system with regionally diverse political economies, one province could threaten to block or weaken national reform efforts.

For these reasons, we should expect public support for climate policy to vary spatially. However, existing data on Canadian climate opinions have been restricted to the national and provincial levels, masking this consequential variation in public beliefs. This research article helps fill this gap by extending methods advanced by [15] in their analysis of local climate opinions in the United States. We use multi-level regression and post-stratification (MRP) to provide the first detailed opinion map of climate and energy opinions in a non-US setting. In doing so, we examine how Canadian perceptions of climate change, as well as policy preferences for carbon taxes and carbon cap and trade, are distributed across the country. Just as scientists have developed disaggregated maps of climate impacts, we present geographically-resolved data on Canadian climate beliefs to inform decision makers at the national, provincial and local levels.

At the national level, previous work has found that 81% of Canadians believe climate change is happening but only 47% think climate change is caused *mostly* by human activities [16, 17]. Relative to the American public then, Canadians are significantly more likely to perceive solid evidence of a global warming trend, and this difference has remained stable over time. However, in this article we find that such aggregate assessments mask important local variation within the Canadian federation. In Nova Scotia for example, we report that 87% say climate change is happening, whereas 66% report this belief in Saskatchewan; within federal electoral districts, percentages vary from 56% in the Souris–Moose Mountain district in Saskatchewan, to 91% in the district of Halifax, Nova Scotia.

Further, we find broad support for climate policy action across Canada. Majorities of the public in every federal electoral district (riding) support an emissions trading scheme, reaching a high of 78% in the Quebec district of Laurier–Sainte Marie. However, support for carbon taxes is more geographically differentiated, ranging from a low of 35% in the Northern Alberta district of Fort-McMurray–Cold Lake to a high of 70% in the Montreal-area district of Outremont. While there is plurality support for carbon taxes in a majority of Canadian federal electoral districts, strong public support is concentrated in British Columbia and urban Canada.

In an effort to support analysis of Canadian climate policy and Canadian climate risks, these new opinion data are made freely available to researchers and members of the public at http://www.umontreal.ca/climat/. A mirror is available at http://environment.yale.edu/ycom/canada/2016/map/. Additional model results are also provided as supporting information in our S1 File.

## Methods

### Data

Our new opinion models build from a series of probability-based, regionally stratified and nationally representative Canadian survey samples. Four separate waves of the *Canadian Surveys on Energy and the Environment* were undertaken in 2011 (n = 1214), 2013 (n = 1502), 2014 (n = 1401) and 2015 (n = 1014). All interviews were administered by Computer Assisted Telephone Interviewing (CATI) using Random Digit Dialing (RDD) and were conducted in either English or French by professional CATI service providers, Léger (2011, 2013 and 2014) and Elemental Data Collection (2015). These dual-frame samples included landline and cell phone listings from the 10 Canadian provinces, and excluded phone sample records from the less populated northern territories. Due to the relatively small proportion of people living in Canada’s territories, public opinion research typically excludes these residents. We obtain a combined response rate of 8% averaged across samples. This estimate relies on the American Association of Public Opinion Research method for calculating response rates (AAPOR RR3). According to a Pew Research Center study [18] a general decline in response rates can be observed across nearly all types of surveys in the United States as well as in other countries. In 2012, the typical response rate for a Pew RDD telephone survey was 9%. Some experiments have found limited evidence of non-response bias resulting from this decline in telephone response rates [19, 20]. Additionally, inclusion of cell phones in our 2013, 2014 and 2015 samples tends to lower average response rates (because completed cell phone surveys are more difficult to obtain); however, cell phone coverage also ensures better representation of younger demographics that are less inclined to have a landline phone number. This dual-frame sampling strategy has become a standard methodology in the survey research industry to minimize non-coverage bias and maximize sample representativeness over the past 10 years [21, 22].

Respondents were geocoded using self-reported postal codes, which were then matched to latitude and longitude coordinates created from the June 2013 Postal Code Conversion File (PCCF), available from Statistics Canada. In cases where respondents did not provide their postal code, postal codes contained in the CATI sample files were used. Where necessary, secondary matching was performed using the first three characters of the six-character Canadian postal code (known as Forward Sortation Areas, or FSAs), which were obtained in the original telephone sample files. We achieve an overall match rate of 87.8% using the full self-reported six-character Canadian postal code, with a further 9.7% of respondents geocoded using the first three characters of the postal code and matching to FSA centroids created from the 2011 FSA map shapefile from Statistics Canada. In a handful of exceptions, respondents were geocoded by matching to either the Census subdivision (1.2%), Census division (0.9%), or postal district (the first character of the postal code, 0.4%). These procedures allow for latitude-longitude coordinates to be assigned to all survey respondents. Finally, the federal electoral district of each respondent was identified by overlaying Elections Canada shapefiles of Canadian electoral districts (using the 2013 Representation Order, i.e., the electoral districts first used nationally in the 2015 federal general elections) on respondent latitude-longitude coordinates.

Local public opinion models are most accurate when geographic-level covariates are chosen that maximize the ratio of inter-unit to intra-unit opinion variation [23]. Our study uses covariates that have broad predictive power in other studies (e.g. political party vote share) with variables that are more closely associated with climate and energy issues. Overall, our estimation methodology closely follows the variables deployed in the U.S. context by [15]. We estimate public opinion at two scales: the province and the 2015 federal electoral district, also called a riding.

Our primary covariate data come from the 2011 Canadian National Household Survey. For each province and district, we construct variables for the fraction of residents that: drive to work; belong to the Christian faith; and are employed in the North American Industry Classification System (NAICS) 21 industries, which focus on fossil fuel, natural gas and mineral mining and extraction. We also merge election results data from the 2011 Canadian federal election to our data set. We use Elections Canada data that reprojects 2011 election results onto the 2013 electoral district representation order. In other words, this file redistributes all votes from the 2011 election into current district boundaries. We explored the use of per capita carbon emissions in the model; however, this data did not provide marginal predictive power above and beyond data on NAICS 21 employment. To measure per capita carbon emissions, we developed a custom data set using the FFDAS 2010.01°grid raster to estimate the point source carbon emissions for each Canadian federal electoral district and province.

To support the post-stratification stage of our MRP models, we obtained custom crosstabs from Statistics Canada; these data provide language by education by gender population count crosstabs from 2011 census data for each 2013 federal electoral district and each Canadian province. We calibrated the categorization in our climate opinion survey data with the census cross tabs by coding language into three categories (English, French, Other), gender into two categories (Male, Female), and education into four categories (Less than high school graduate, High school graduate, Some college or technical school, college or graduate degree).

### Model Specification

Our methods follow [15]’s approach in their study of the distribution of U.S. climate and energy opinions. We deploy a two-stage estimation procedure. First, we use multi-level regression to estimate the relationship between individual and geography-level covariates and specific opinions. Second, we weight the estimated opinions of individual demographic-geographic types according to their frequency distribution across geographic units of interest. Together, this multi-level regression and post-stratification (MRP) approach enables opinion estimation in different political geographies. Public opinion scholars have extensively elaborated and validated this methodology [23–26].

During the *multi-level regression* step, we estimate the relationship of individual and geography-level covariates to a specific climate and energy opinion, h, for a given individual, i, represented by *y*_*h*[*i*]_. For example, we could estimate the relationship between belief that climate change is occuring (h), and a number of individidual demographic characteristics (i). For clarity, we specify the model here for a single opinion only, dropping the indexing over h. At the individual-level, we thus have:

$$\begin{array}{c} Pr\left(y_{i}=1\right)=logit^{-1}\left(\gamma_{0}+\alpha_{j[i]}^{language}+\alpha_{k[i]}^{education}+\alpha_{l[i]}^{gender}+\alpha_{m[i]}^{time}+\alpha_{p/d[i]}^{geography}\right) \end{array}$$

$$\begin{array}{c} \alpha_{j}^{language}\;∼\;N\left(0,\sigma_{language}^{2}\right),\;for\;j=1,...,3 \end{array}$$

$$\begin{array}{c} \alpha_{k}^{education}\;∼\;N\left(0,\sigma_{education}^{2}\right),\;for\;k=1,...,4 \end{array}$$

$$\begin{array}{c} \alpha_{l}^{gender}\;∼\;N\left(0,\sigma_{gender}^{2}\right),\;for\;l=1,2 \end{array}$$

$$\begin{array}{c} \alpha_{m}^{time}\;∼\;N\left(0,\sigma_{time}^{2}\right),\;for\;n=1,...,4 \end{array}$$

Each variable is indexed over individual i and over response categories j,k,l and m for language, education, gender, and time variables respectively. The geography variable is flexible, indexing either provinces (p) or federal electoral districts (d) depending on which geographic level is being modeled. The *time* variable indicates the survey wave of particular respondents in the model. Including this *time* variable allows us to generate estimates tailored to the most survey time period even as we pool across multiple time periods.

For district models, we model the geography-level term as:

$$\begin{array}{c} \alpha_{d}^{district}\;∼\;N(\alpha_{z[r]}^{province}+\gamma^{drive}\cdot drive_{s}+\gamma^{christian}\cdot christian_{s}+\gamma^{industry}\\ \cdot industry_{s}+\gamma^{convote}\cdot convote_{s},\sigma_{district}^{2}),\;for\;d=1,...,338 \end{array}$$

where, for each political geography, *drive* is the fraction of residents who report driving a car, truck or van to work, *christian* is the fraction of residents who report themselves as belonging to the Christian faith, *industry* is the fraction of residents who are employed by mining, quarrying, and oil and gas extraction industries, and *convote* is the fraction of votes for the Conservative Party during the 2011 federal election.

We exclude territories because of a low respondent pool from territories in our combined national survey sample. Therefore, our province variable in the district specification is further modeled by:

$$\begin{array}{c} \alpha_{z}^{province}\;∼\;N\left(0,\sigma_{province}^{2}\right),\;for\;j=1,...,10 \end{array}$$

By contrast, our province-specification has a similar form to our district specification, except it omits the first *α*_*z*_[*d*]^*province*^ term altogether, has variance $\sigma_{district}^{2}$, and is indexed over *p* = 1, …, 10 All models are run using the ‘glmer’ function, available in the lme4 package in R [27].

In the *post-stratification* step, we first produce a table of population counts for each individual demographic type (language, gender, education) for each geographic unit (province or district). Given that the cross-tabs summarize these three variables across 9 total levels, each geographic subunit is associated with 24 individual demographic counts. For example, one cross-tab is the number of high-school educated men with English as their first language living in the Halifax West district. Our multi-level regression model produces an estimated level of opinion for each demographic type in each political geography. With 10 provinces at 335 districts, and 24 population types, we thus generate 240 unique provincial and 334∗24 = 8016 district estimates. There are 338 total federal electoral districts in the 2015 representation order. We exclude the three territories and Labrador because we do not have any respondents from these three territories, and only one respondent from Labrador.

Next, we estimate an opinion’s distribution in a given geographic area. We weight the multi-level regression estimates for each population type’s opinion estimate according to that population type’s proportion of the total population in that unit. Formally, let *ϑ*_*w*_ describe the estimated opinion of each unique demographic-geography type, indexed over cell *w*, and let *N*_*w*_ give the population count for that cell. Our MRP opinion estimates for a given geography can be calculated over the set of all provinces or districts *g*:

$$\begin{array}{c} y_{province(district)}^{mrp}=\frac{\Sigma_{w\epsilon g}N_{w}\vartheta_{w}}{\Sigma_{w\epsilon g}N_{w}} \end{array}$$

## Model Validation

Internal validation was performed to test the accuracy and reliability of our model estimates in the Canadian context. This technique follows a cross-validation procedure described in [28] and used in [15]. In repeated simulations, subsamples of varying sizes were randomly selected from a large-population province or district and used to simulate the samples of smaller provinces or districts. We then compare MRP model estimates on these subsampled data sets to estimates from the full data set. For example, we take the largest province, Ontario, and through random subsampling reduce the number of respondents in Ontario (n = 1642) to the sample sizes of smaller provinces, from Alberta (n = 750) to Prince Edward Island (n = 21). We repeat the subsampling procedure across 99 simulations, for the four largest provinces (Ontario, Quebec, British Columbia, and Alberta), and for our five dependent variables.

We also performed crossvalidation at the district level in the same manner. In this case, we take advantage of sampling variation in the original surveys and validate using districts with the largest number of survey respondents. These districts were slightly oversampled in the original surveys and are geographically and demographically diverse. At the district level we use five districts ranging from n = 29 to n = 68 respondents (Courtenay–Alberni, BC; Selkirk–Interlake–Eastman, MB; Manicouagan, QC; Sherbrooke, QC; and Eglinton–Lawrence, ON). We randomly reduce the number of respondents in each district to 25, 20, 15, 10, and 5 respondents. We repeat this subsampling across 99 simulations for each of the five dependent variables. We compare these cross-validation estimates to survey subsamples for each geographic area. We repeat this for samples post-stratified by gender, educational attainment, and language to account for possible demographic non-representativeness of the national sample within smaller geographic areas.

**Fig 1:**
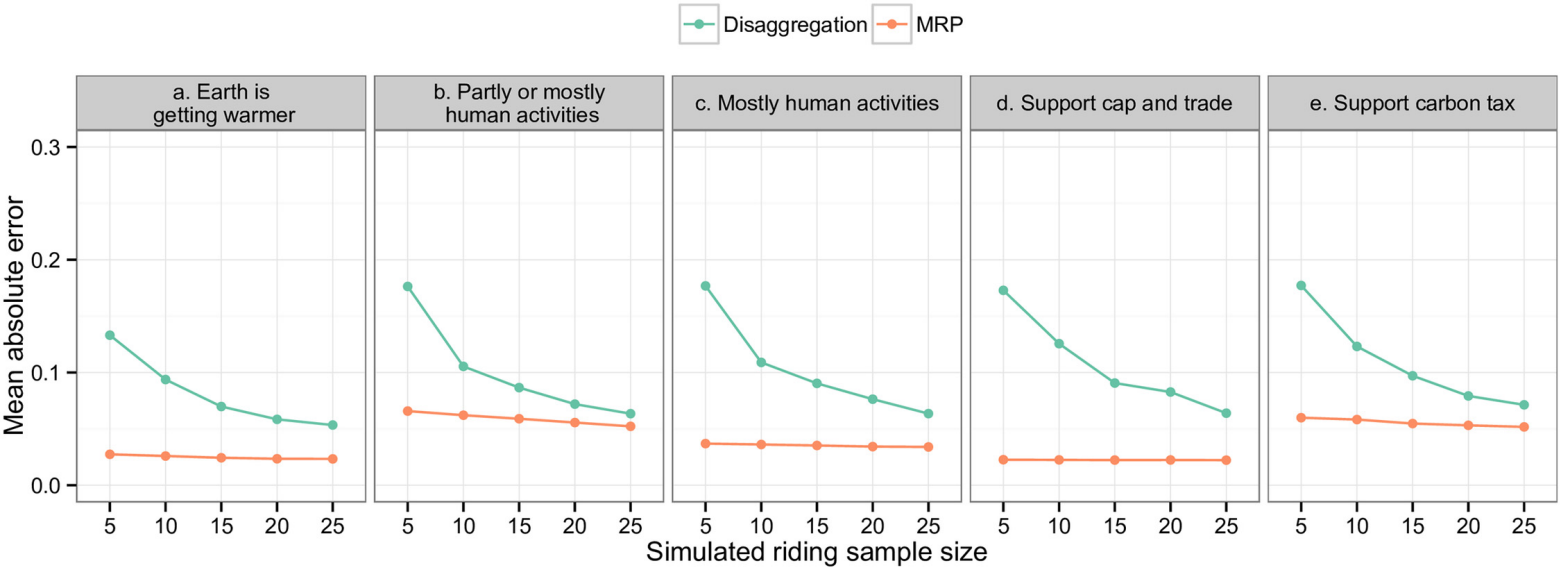
Cross-validation compared to disaggregation across five simulated sample sizes (n = 99 simulations). Figure shows mean absolute error across the MRP results and disaggregation of the full sample at the district level. The analysis is for five variables: belief that climate change is happening, belief that cliamte change is at least partly human-caused, belief that climate change is mostly human-caused, support for cap and trade, and support for a carbon tax. For each variable, we average across five districts.

The predictive ability of the MRP model can be assessesed by comparing mean absolute errors across each simulated sample size. We also calculate the mean absolute error from raw disaggragated projections for each geographic area and subsample size for comparison against the MRP estimates. Fig 1 illustrates the performance of the MRP model at the smaller district level across our five variables. MRP consistently outperforms disaggregation for each variable at the district level. As expected, the mean absolute error of both the MRP and disaggregation techniques decreases as we increase the simulated sample size of each district. MRP vastly outperforms disaggregation in areas with smaller populations, which mirrors findings from previous work in the U.S. [15]. Results from the crossvalidation tests at the province level indicate that MRP performs similarly to disaggregation (results provided in the online SI). Disaggregating a large set of national survey data across the 10 provinces produces results with similar accuracy to our MRP model. However, MRP also carries an additional advantage over disaggregation at this level–it is able to account for changes in opinion over time, unlike simple disaggregation of survey data collected over multiple years.

Finally, margins of error were calculated from 999 bootstrap simulations of the multi-level regression models at the 95% confidence level. Margins of error averaged ±6 percentage points for the provincial-level estimates and ±7 percentage points for the district-level estimates.

## Results

Our results reveal, for the first time, the diversity of Canadian climate and energy opinions at the local level. Additional model results are reported in supporting information provided as part of our S1 File. A strong majority of Canadians believe that climate change is happening. However, national opinion levels mask substantial variation across the country. We summarize districts with exceptionally high and somewhat lower beliefs in Table 1. The strongest levels of climate change belief exist in coastal BC, Quebec, Nova Scotia as well as in urban areas across the country. Belief in climate change is somewhat lower in rural Canada, particularly across the Prairies. However, despite this variation, a majority of Canadians in each federal electoral district believe that climate change is happening. Further, in over 97% of districts (n = 324), belief levels are over 60% and in just under 89% of districts belief levels are over 70%. Note that we only estimate belief levels in 334 of Canada’s 338 electoral districts. These figures exclude the Northern territories and the Newfoundland district of Labrador. At the extreme, belief in climate change exceeds 90% of the public in the Quebec district of Laurier-Sainte-Marie, the district of Vancouver East, and the district of Halifax.

**Table 1:** Top 10 and Bottom 10 Canadian Federal Electoral Districts, % Population who believe that Earth is getting warmer.

Top 10 Districts	Bottom 10 Districts
Brampton East (ON)	Battle River–Crowfoot (AB)
Dartmouth–Cole Harbour (NS)	Bow River (AB)
Halifax (NS)	Foothills (AB)
Laurier–Sainte-Marie (QC)	Fort McMurray–Cold Lake (AB)
Outremont (QC)	Lakeland (AB)
Papineau (QC)	Peace River–Westlock (AB)
Rosemont–La Petite-Patrie (QC)	Portage–Lisgar (MB)
Surrey–Newton (BC)	Red Deer–Lacombe (AB)
Terrebonne (QC)	Souris–Moose Mountain (SK)
Vancouver East (BC)	Yorkton–Melville (SK)

*Note* Districts listed alphabetically by category.

**Fig 2:**
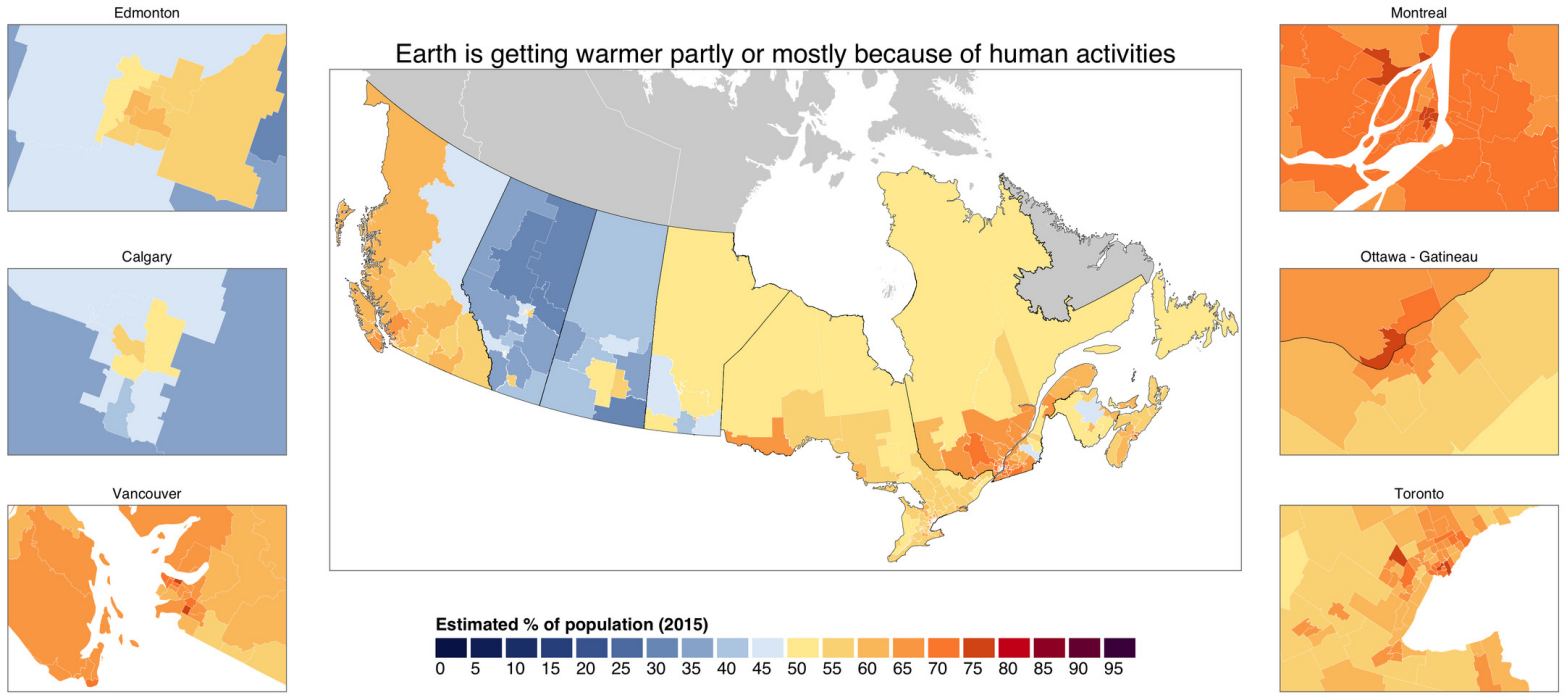
Estimates of Canadian public belief that the Earth is getting warmer partly or mostly because of human activities. Estimated at the federal electoral district (riding) level.

Canadians hold more variable beliefs about climate change’s causes. Fig 2 visualizes the estimated proportion of Canadians who believe that humans contribute to observed climate change. This variation has both a regional component and a rural-urban component. The figure insets highlight higher levels of belief in the six largest urban areas of Canada. Opinions in these urban areas contrast sharply with estimated beliefs in surrounding rural areas. Regionally, belief that climate change is human-caused is lowest in the more greenhouse gas intensive parts of Canada, including parts of northern Alberta and Saskatchewan where oil sands developments are located. In other words, places that are more significantly contributing to climate change show lower beliefs that humans are the cause. At the same time, urban districts in Alberta, including Calgary and Edmonton, have publics who more closely match publics in Ontario, Quebec, or British Columbia.

**Fig 3:**
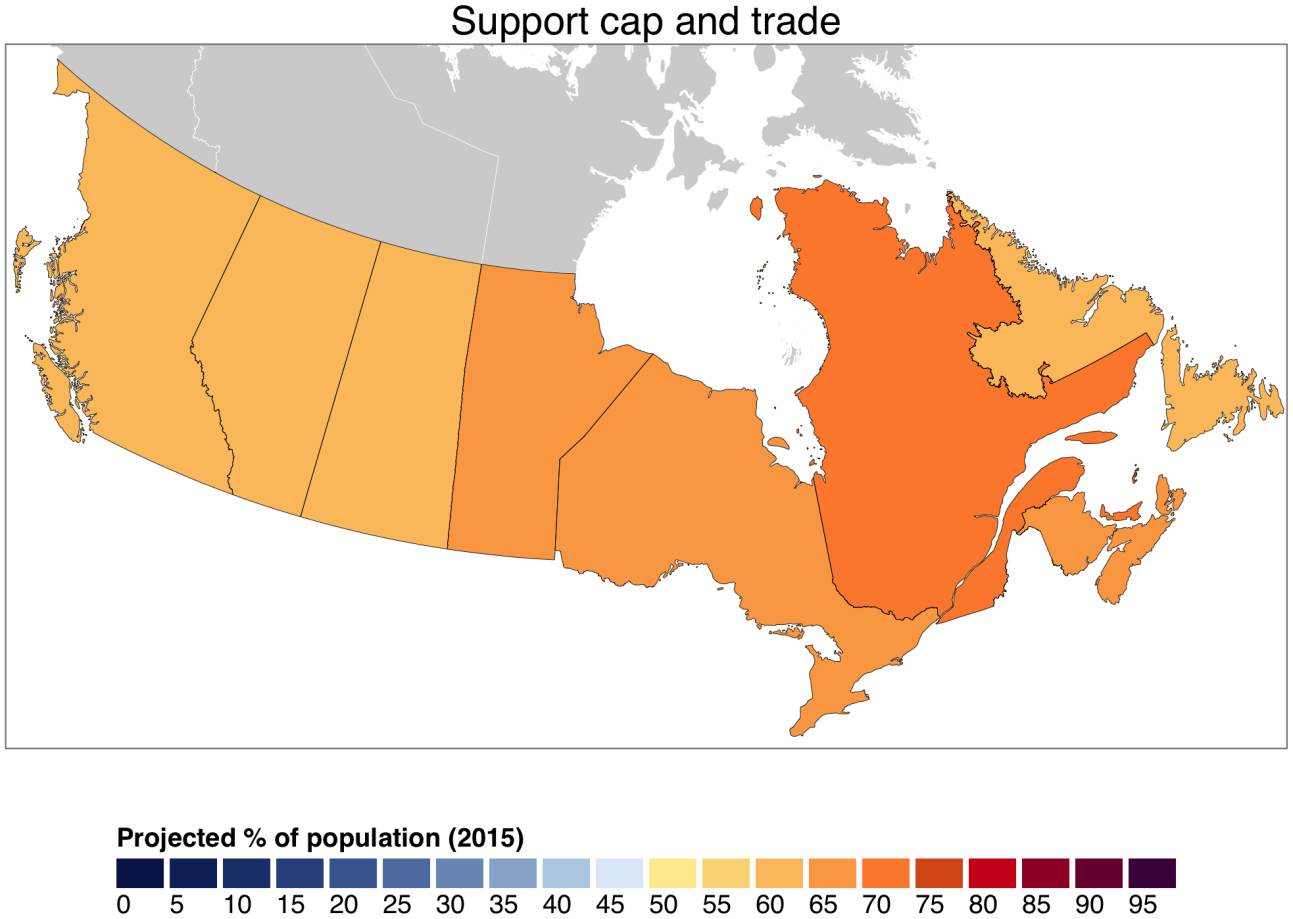
Estimates of Canadian public support for emissions trading at the provincial level.

Despite this variation in core beliefs about climate change, we find widespread public support for climate policies. Support is greatest and most consistent for emissions trading. Fig 3 depicts majority support for carbon cap and trade across each Canadian province. At the district level, support for cap and trade is thinner in rural Northern Alberta than in other areas, such as urban Southern Quebec. However, the overall pattern is clear: there is majority support for emissions trading in *every* Canadian district. The lowest support is 56% in the Alberta district of Battle-River-Crowfoot. Support exceeds 66% in over half of Canadian districts, reaching a high of 75% in the Montreal-area Laurier-Sainte-Marie district.

**Fig 4:**
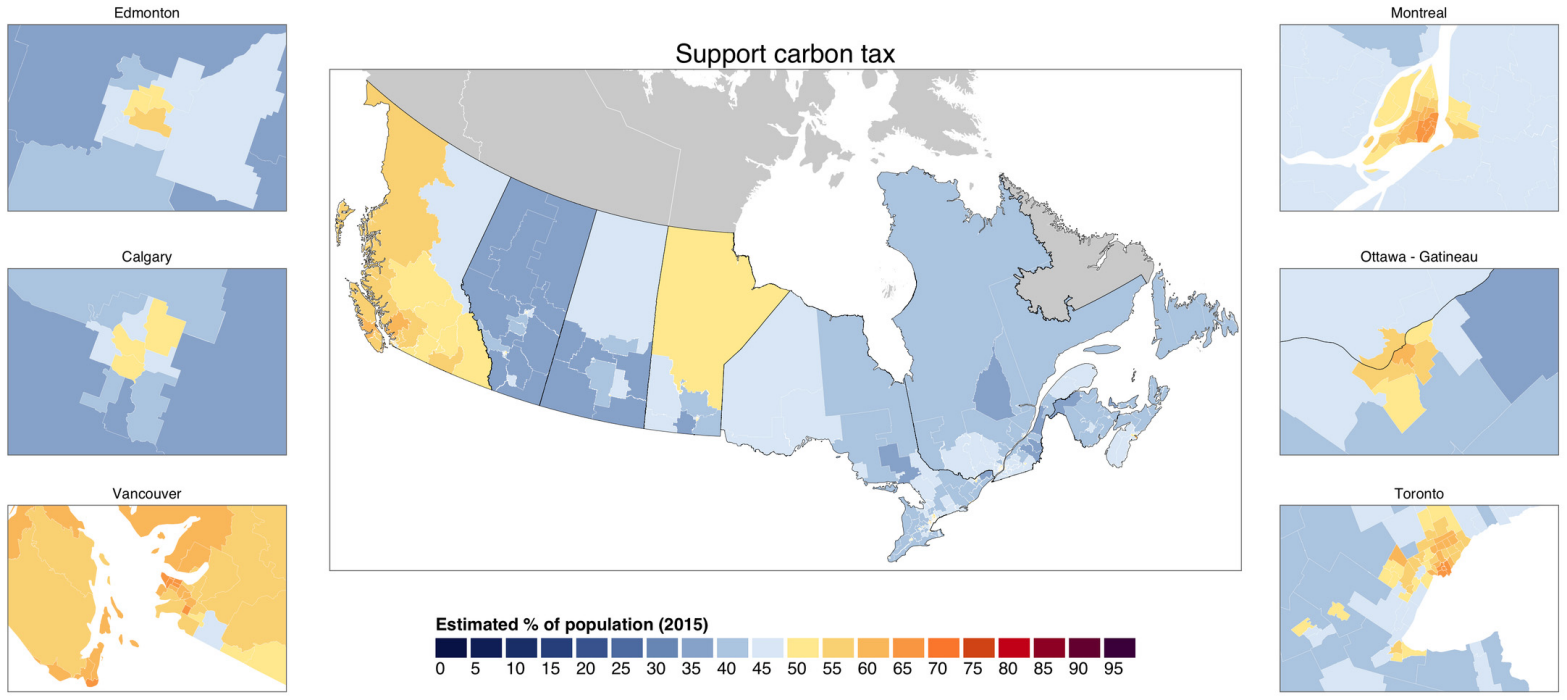
Support for a carbon tax in Canada at the federal electoral district (riding) level.

We find larger variation in support for a carbon tax across the country in Fig 4. At the national level, we estimate support for carbon taxation at 49%, just below a majority, with opposition at 44%. However, many parts of rural Canada only show strong minority support for carbon tax reforms. In 308 of 334 districts (90%), support for a carbon tax is above 40%. And there is majority support in 141 districts (40%). The largest support is found in the Montreal, Quebec district of Outremont, where we estimate 70% of citizens support a carbon tax. Nonetheless, overall support is geographically concentrated in British Columbia and across urban Canada. The ridings with the highest and lowest support for a Canadian emissions trading scheme are listed in Table 2.

**Table 2:** Top 10 and Bottom 10 Canadian Federal Electoral Districts% Population who Support Carbon Cap and Trade Policy.

Top 10 Districts	Bottom 10 Districts
Ahuntsic-Cartierville (QC)	Battle River–Crowfoot (AB)
Hochelaga (QC)	Bow River (AB)
Laurier–Sainte-Marie (QC)	Cypress Hills–Grasslands (SK)
Notre-Dame-de-Grace–Westmount (QC)	Fort McMurray–Cold Lake (AB)
Outremont (QC)	Lakeland (AB)
Papineau (QC)	Peace River–Westlock (AB)
Rosemont–La Petite-Patrie (QC)	Portage–Lisgar (MB)
Toronto–Danforth (ON)	Red Deer–Mountain View (AB)
University–Rosedale (ON)	Souris–Moose Mountain (SK)
Ville-Marie–Le Sud-Ouest–Île-des-Soeurs (QC)	Yellowhead (AB)

*Note* Districts listed alphabetically by category.

In order to capture a broader range of views on carbon pricing options, we also model levels of public opposition to carbon pricing policies across the country. This allows for an assessment of whether carbon taxation has plurality support–more people supporting a policy than opposing it–at the district level. We find plurality support in just under 60% of Canadian federal electoral districts. These levels are substantially higher than comparable levels of support for carbon taxation in the United States [15]. However, these results again suggest that the Canadian public remains more divided over carbon taxation than emissions trading.

Notably, support for carbon pricing policies is higher in provinces that have already implemented these policies. For instance, support for a carbon tax is highest in British Columbia (57%), where a $10 CDN per tonne of CO2 equivalent carbon tax was introduced in 2008. Today, the BC tax rate is $30 CDN, the highest explicit carbon price in Canada. Similarly, support for emissions trading is highest in Quebec (71%), the first Canadian province to implement a functioning carbon market that took effect in 2013. Subsequent to the joint auction held in 2014, the Quebec cap and trade system is now fully integrated with the California cap and trade program. In this linked market, allowances are currently trading at approximately $12 USD per tonne of CO2 equivalent. Support for emissions trading is extremely high in Quebec, with 8 of the top 10 districts supporting the policy located in the province, as Table 2 shows. Static, cross-sectional opinion data cannot arbitrate whether this public support preceded government policy enactment, or whether it reflects increased public comfort with climate policies after implementation. At a minimum, our public opinion estimates suggest there is little evidence of popular backlash against carbon pricing in Canadian jurisdictions with implemented policies.

**Fig 5:**
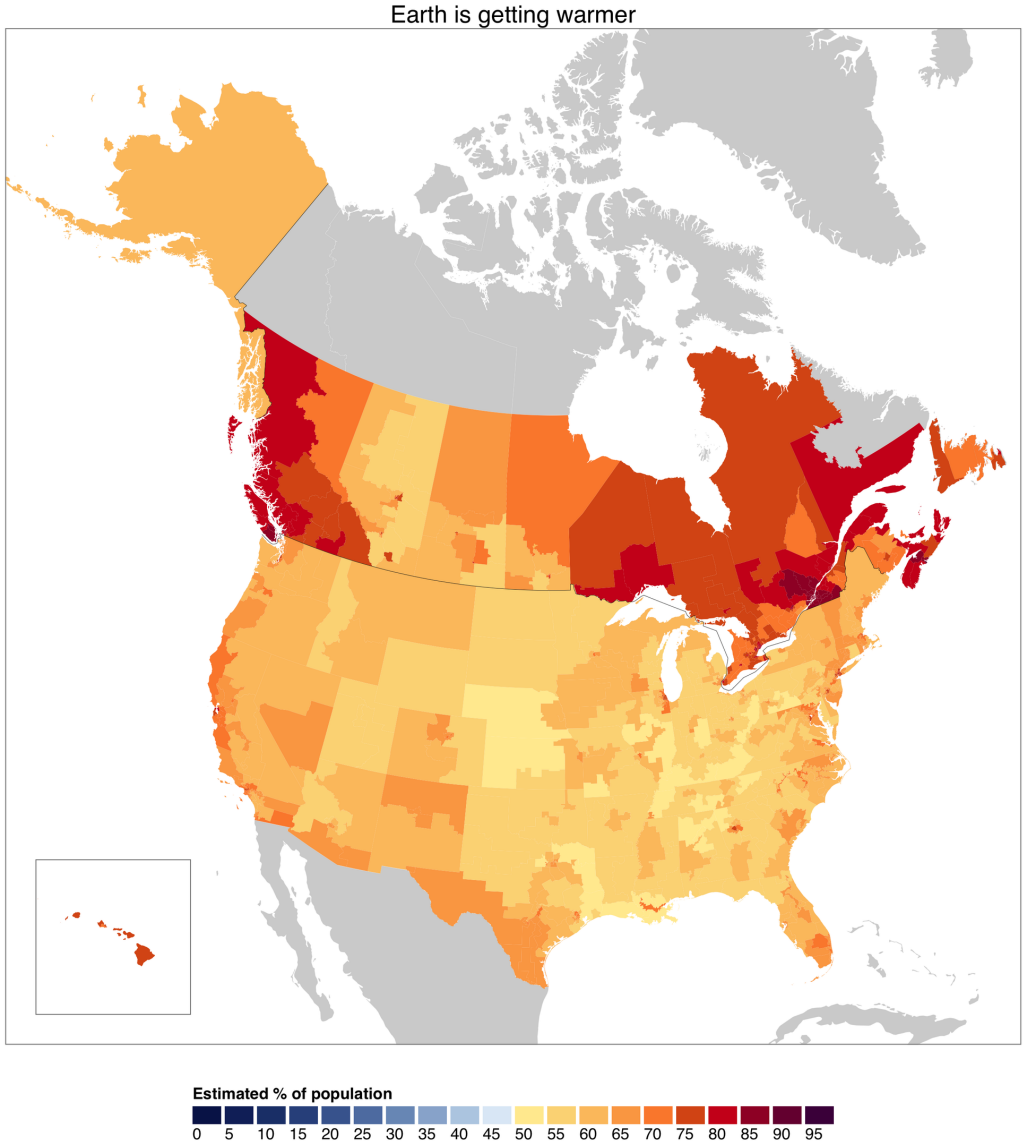
Belief that Earth is getting warmer across North America. Canadian data comes from this new data set at the district (riding) level. US data is presented at the Congressional District level from a data set by [15].

Canadian beliefs that climate change is happening can be compared to the distribution of climate beliefs in the United States. In Fig 5 we merge the Canadian district-level results with U.S. estimates for 2014 at the congressional district level, as measured by the Yale Project on Climate Change Communication and available as part of [15]. These U.S. estimates are available at http://environment.yale.edu/poe/v2014/. Note that the U.S. opinion survey asks the public about their perceptions of ‘global warming,’ while the Canadian survey asks about perceptions of the Earth getting warmer.’ We cannot exclude the possibility that a portion of the differences between Canadian and US opinions estimates is a function of subtle differences in question wording.

Overall, belief in climate change is higher in Canada–the low end of variation in Canadian climate beliefs maps onto the middle end of US variation. Many Canadian districts have levels of belief in climate change that are systematically higher than even the most liberal parts of the United States.

## Conclusion

To date, we have lacked high-resolution social science data to support climate research, policy and planning in Canada. Public opinion data have primarily been national; even accurate provincial data has been sparse. This new public data set will thus fill an important need for high resolution information on public attitudes, perceptions and policy preferences on issues related to climate change and climate mitigation policy.

A window of opportunity has recently opened for climate policy in Canada. After more than a decade of federal inaction, a new government committed to enacting climate policy was elected in late 2015. This new Liberal government aims to develop a plan to price and cap carbon emissions nationally. Given that existing provincial mitigation policy is highly variable in both instrument choice and stringency, the federal government is planning to work with the provinces to strengthen and harmonize policies. Propitiously, a new Alberta government was also elected in 2015. This New Democratic Party government is also committed to enacting new climate policy at the provincial level, phasing out coal electricity by 2030 and capping overall oil sands emissions. Meanwhile, other provinces, including Ontario, Quebec and British Columbia (BC), have already enacted a broad range of climate policy instruments, including phasing out coal, implementing a carbon tax, and working with California on a regional cap and trade system [29–31].

Overall, we find evidence of broad Canadian support for climate policies. The Canadian public overwhelmingly believes the climate is changing, and a majority believe it is at least partly human caused. We find generally high support for emissions trading policies across all districts, with more variation in support for a carbon tax. Even so, over 42% of Canadian districts have majority support for increasing taxes on carbon-based fuels; this is a much higher distribution than seen in the United States and speaks to enhanced public support for action in the short term.

This project also demonstrates the feasibility of efficiently preparing subnational estimates of public climate beliefs outside the United States. Climate change is a global challenge and will require coordinated action by governments across the planet. Efforts to increase the availability of high-quality public opinion data at relevant political scales can supplement geographically-resolved climate and economic risk maps in guiding climate mitigation and adaptation policy responses.

In line with this need, the release of our data set as a supplement to this article will provide an important resource for a range of scientific and policy end-users. It will help support adaptation planning, inform policy decision making across scales in Canada. Most prominently, it provides valuable information about the public’s beliefs regarding one of the most pressing environmental and policy challenges facing Canadians and the world over the coming decade.

## Supporting Information

S1 File The S1 File provides full details of all the underlying survey questions used in this study, additional cross-validation data and additional results. (PDF)
