# Supplementary material for: The Distribution of Climate Change Public Opinion in Canada
Source: PLoS One. 2016 Aug 3;11(8):e0159774. doi: 10.1371/journal.pone.0159774 (PMC4972305; doi:10.1371/journal.pone.0159774)
Supplement: S1 File — (PDF) [file pone.0159774.s001.pdf]

Supplementary material: The distribution of climate change public opinion in Canada

May 18, 2016

1 S1 Appendix: Survey details

Model estimates in the maps were derived from public responses to the following survey questions. The response categories for many questions were collapsed into a single variable for mapping. For example, for the question measuring how much respondents support a carbon tax, 'strongly support' and 'somewhat support' were combined into a single measure of 'Support.' Likewise 'Somewhat oppose' and 'Strongly oppose' were combined into a single measure of 'Oppose.' We differentiate between individuals who believe that the Earth is getting warmer *mostly* because of human activity, and the larger set of people who believe the Earth is getting warmer *mostly or partly* because of human activity. Finally, we do not estimate non-opinions (i.e. "Not sure" and "Refused" responses) measured by these survey questions.

Earth is getting warmer

From what you've read and heard, is there solid evidence that the average temperature on earth has been getting warmer over the past four decades?

- Yes
- No
- Don't Know (volunteered)

Earth is getting warmer because of human activity

[If yes, solid evidence] Is the earth getting warmer mostly because of human activity such as burning fossil fuels or mostly because of natural patterns in the earth's environment?

- Human Activity

- Natural Patterns
- Combination (volunteered)
- Not sure / Refused (volunteered)

Support a cap and trade system

There is a proposed system called cap and trade where the government issues permits limiting the amount of greenhouse gases companies can put out. If a company exceeds their limit, they will have to buy more permits. If they don't use all of their permits, they will be able to sell or trade them to others who exceed their cap. The idea is that companies will find ways to put out less greenhouse gases because that would be cheaper than buying permits.

Do you strongly support, somewhat support, somewhat oppose or strongly oppose this type of system for your province?

- Strongly support
- Somewhat support
- Somewhat oppose
- Strongly oppose
- Not sure / Refused (volunteered)

Support increasing taxes on carbon based fuels

Another way to lower greenhouse gas emissions is to increase taxes on carbon based fuels such as coal, oil, gasoline and natural gas. Do you strongly support, somewhat support, somewhat oppose or strongly oppose this type of system?

- Strongly support
- Somewhat support
- Somewhat oppose
- Strongly oppose
- Not sure / Refused (volunteered)

2 Province-level validation tests

Figure S1 provides the results of province-level cross-validation tests. See the main text for methodological details.

3 Additional results at province and riding geographies

Figures S2 through S6 provide full results at the province level. Figures S7 through S11 provide full results at the electoral district (riding level)

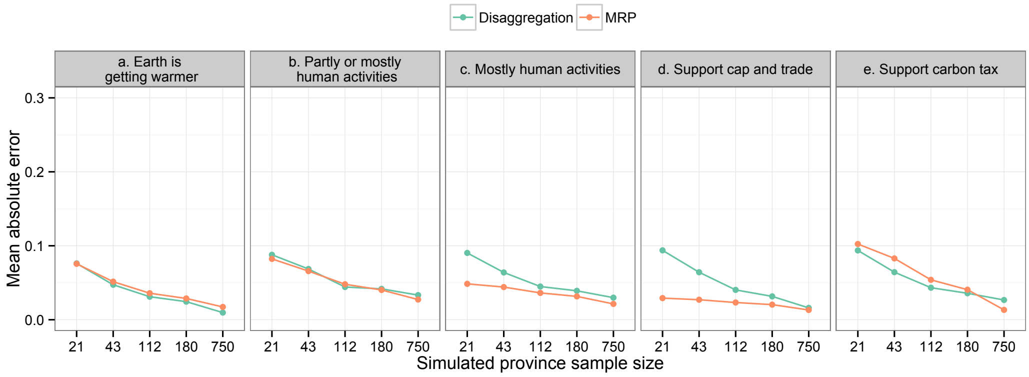

Figure 1: (**S1**) Cross-validation compared to disaggregation across five simulated sample sizes at the province level (n = 99 simulations). Figure shows mean absolute error across the MRP results and disaggregation of the full sample. The analysis is for five variables: belief that climate change is happening, belief that climate change is at least partly human-caused, belief that climate change is mostly human-caused, support for cap and trade, and support for a carbon tax. For each variable, we average across the four largest provinces: Ontario, Quebec, British Columbia, and Alberta.

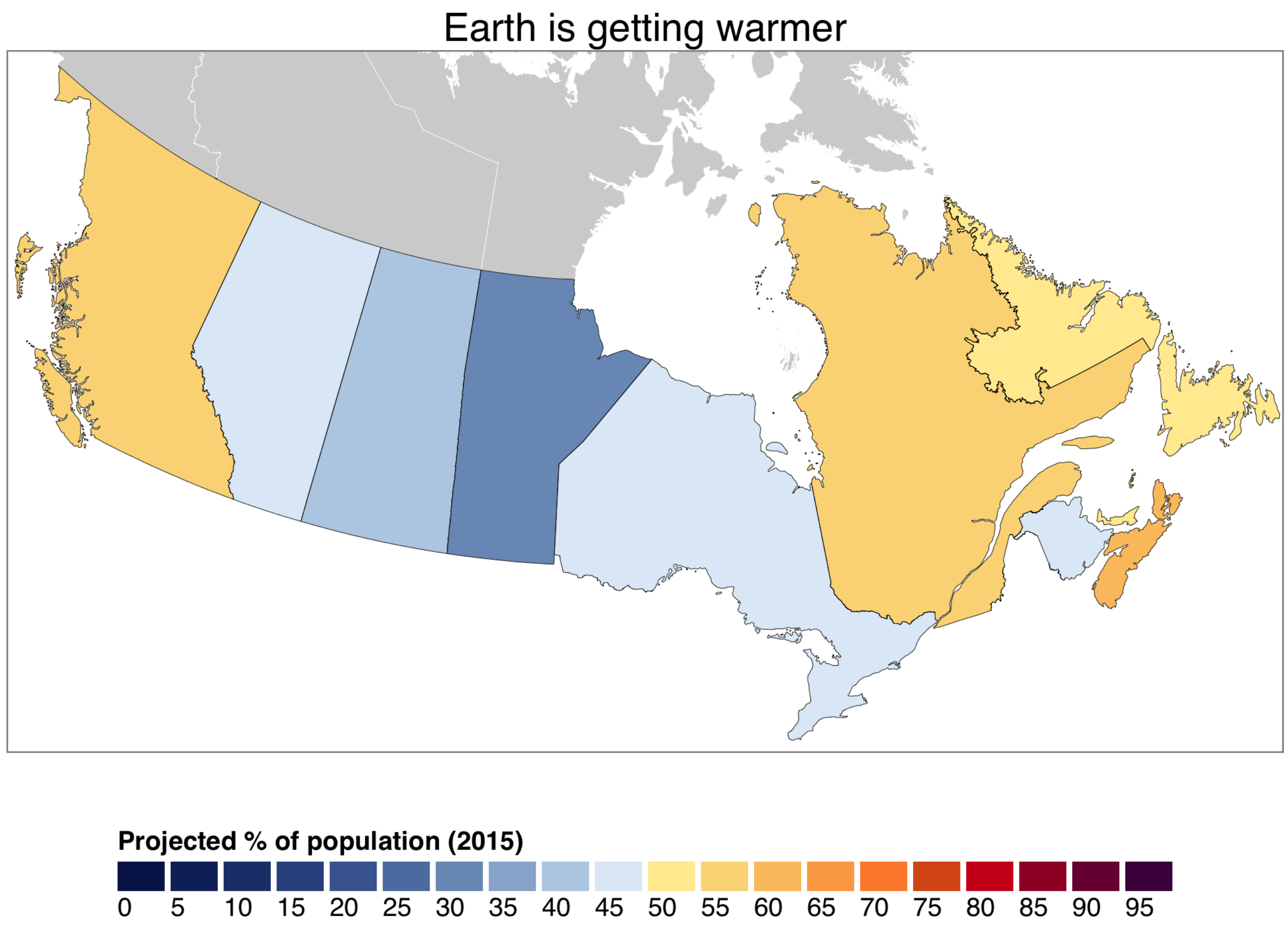

Figure 2: (**S2**) Belief that Earth is getting warmer at the province level.

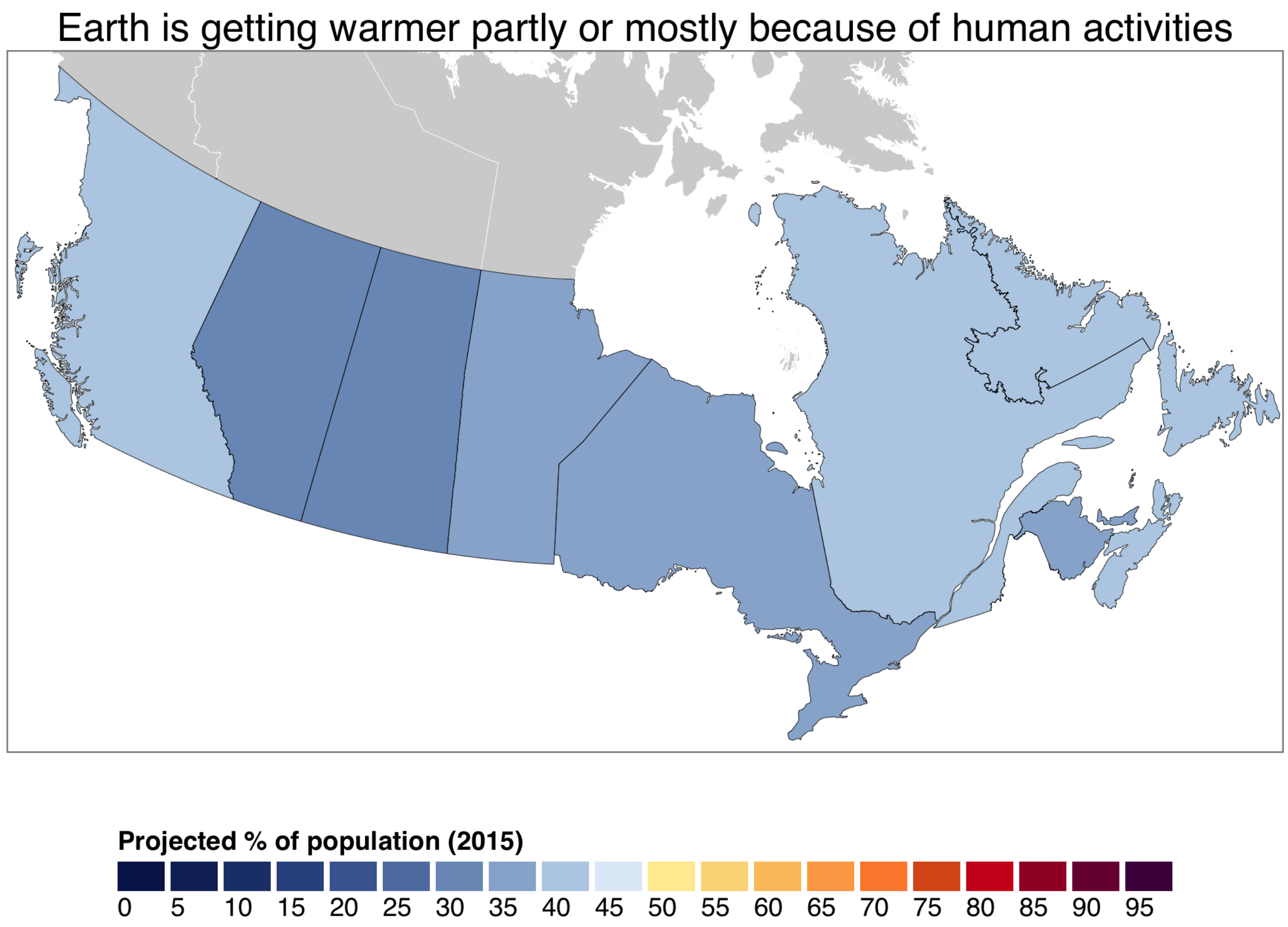

Figure 3: (**S3**) Belief that Earth is getting warmer partly or mostly because of human activities at the province level.

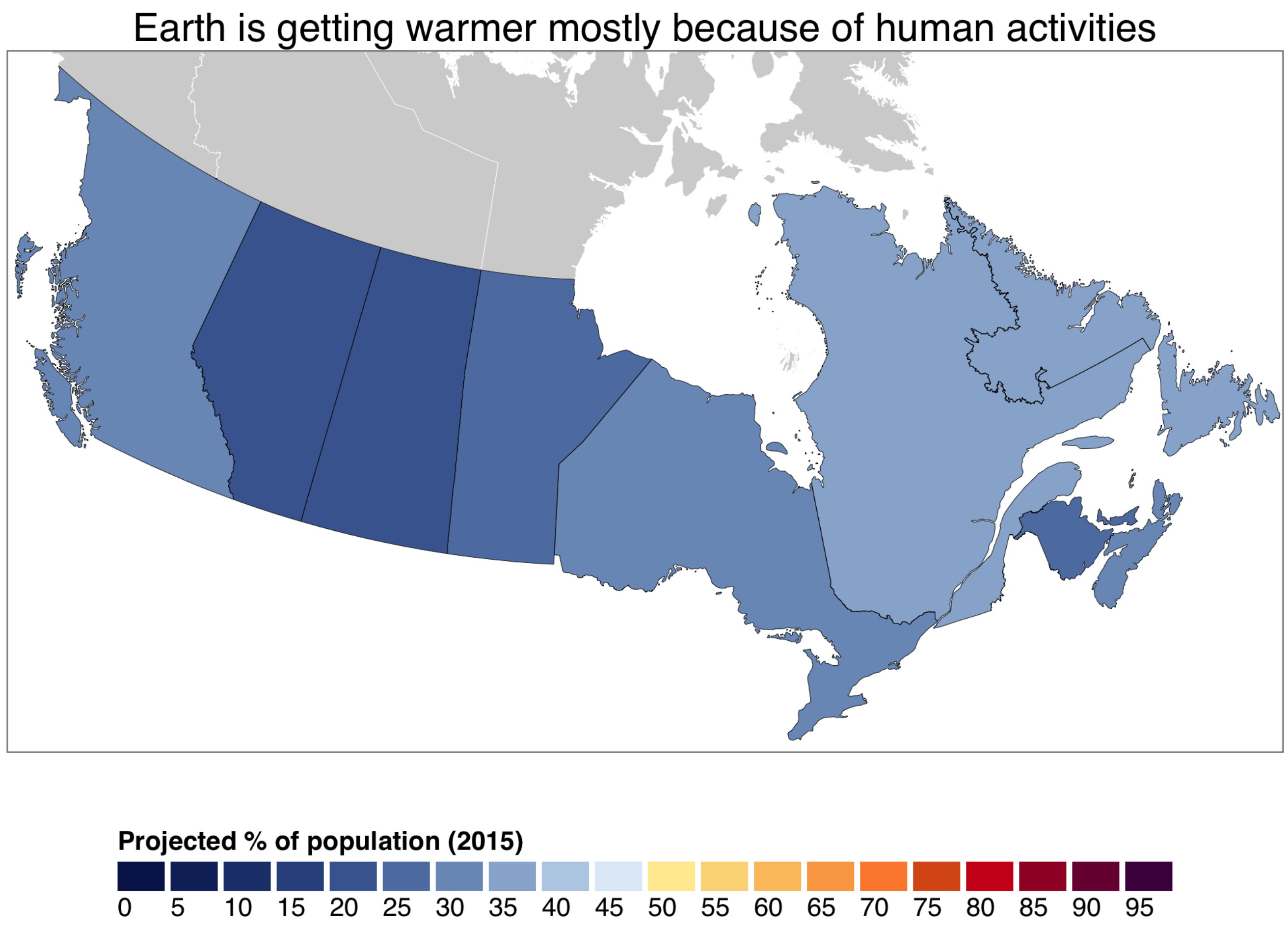

Figure 4: (**S4**) Belief that Earth is getting warmer mostly because of human activities at the province level.

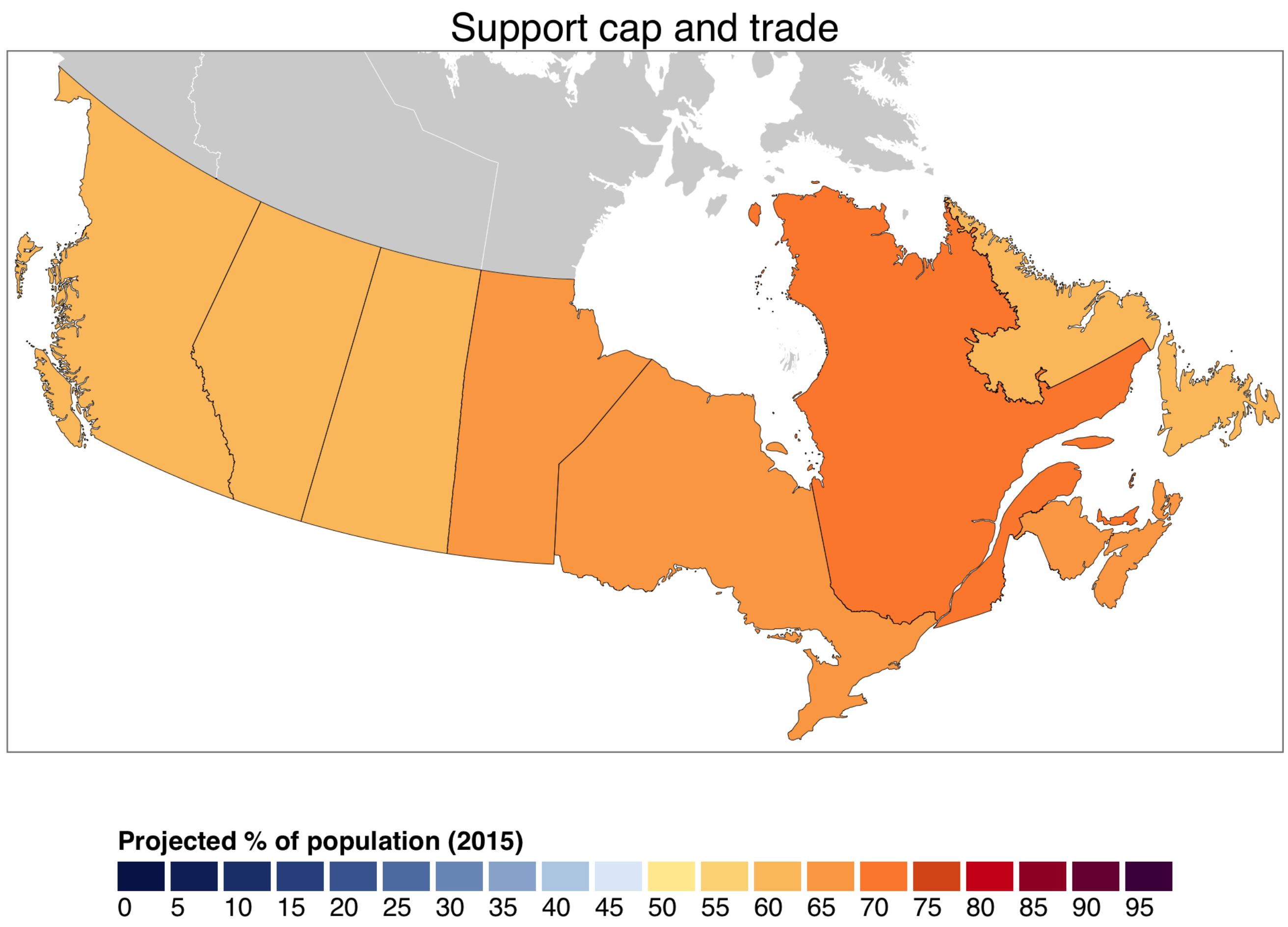

Figure 5: (**S5**) Support for cap and trade at the province level.

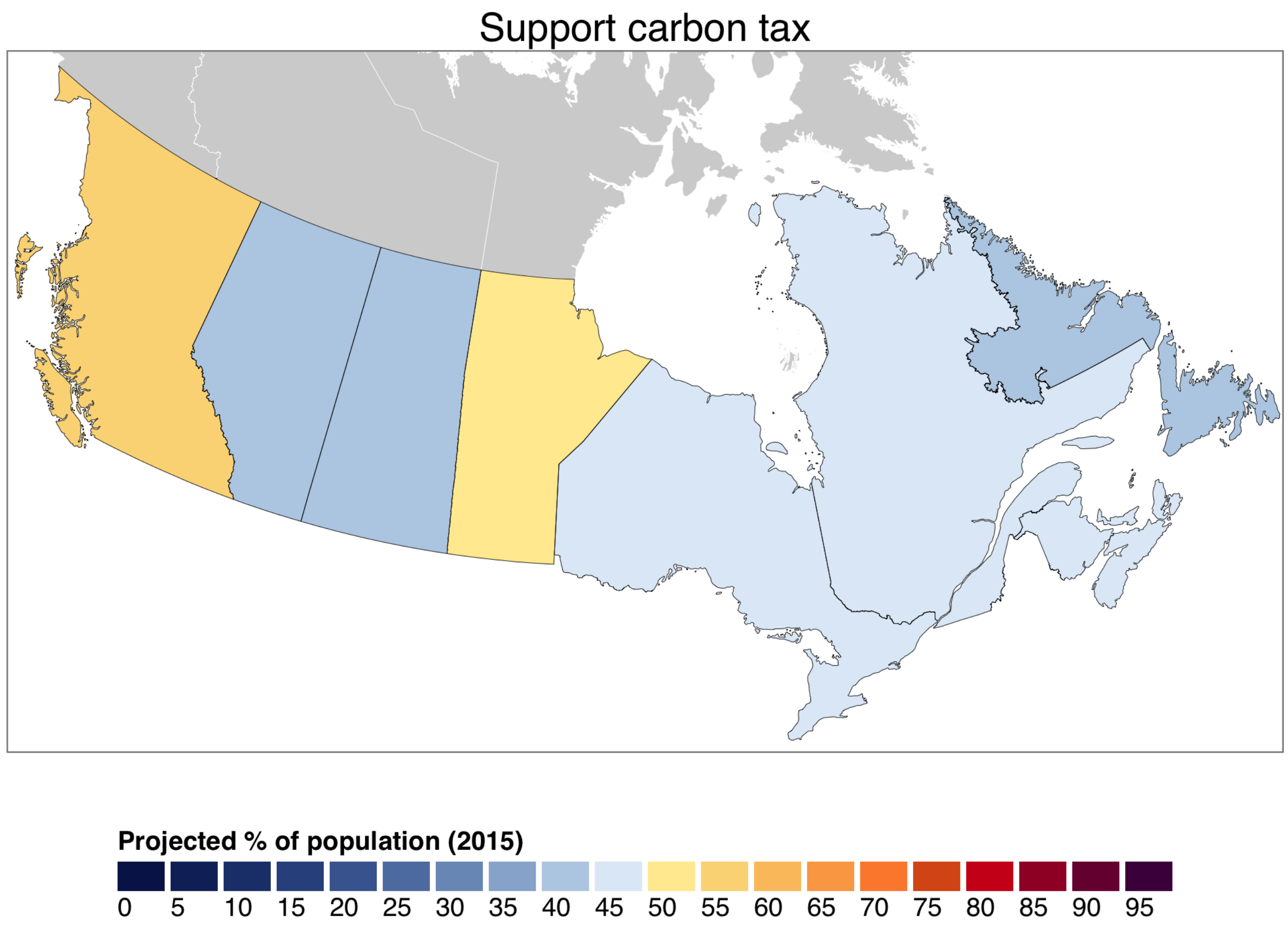

Figure 6: (**S6**) Support for carbon tax at the province level.

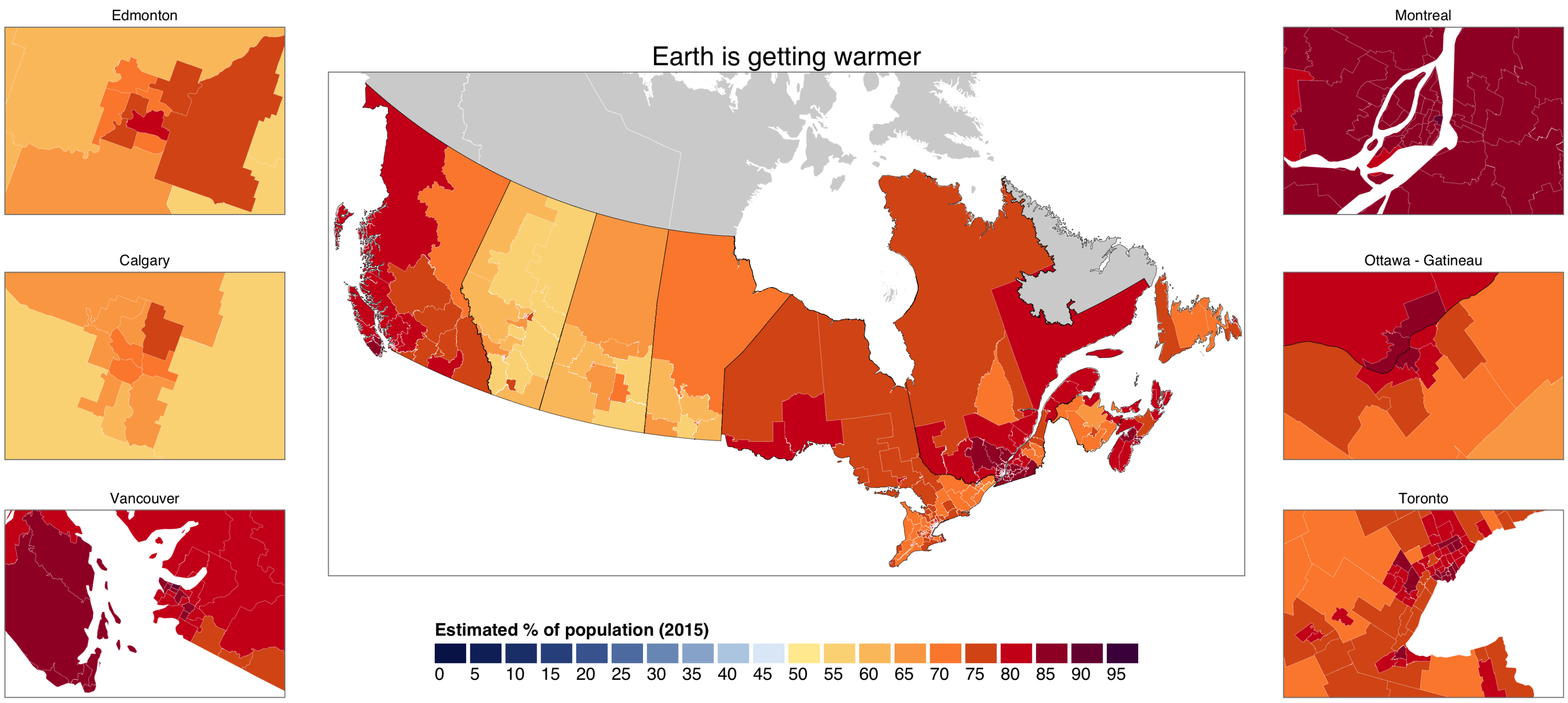

Figure 7: (**S7**) Belief that Earth is getting warmer at the federal electoral district (riding) level.

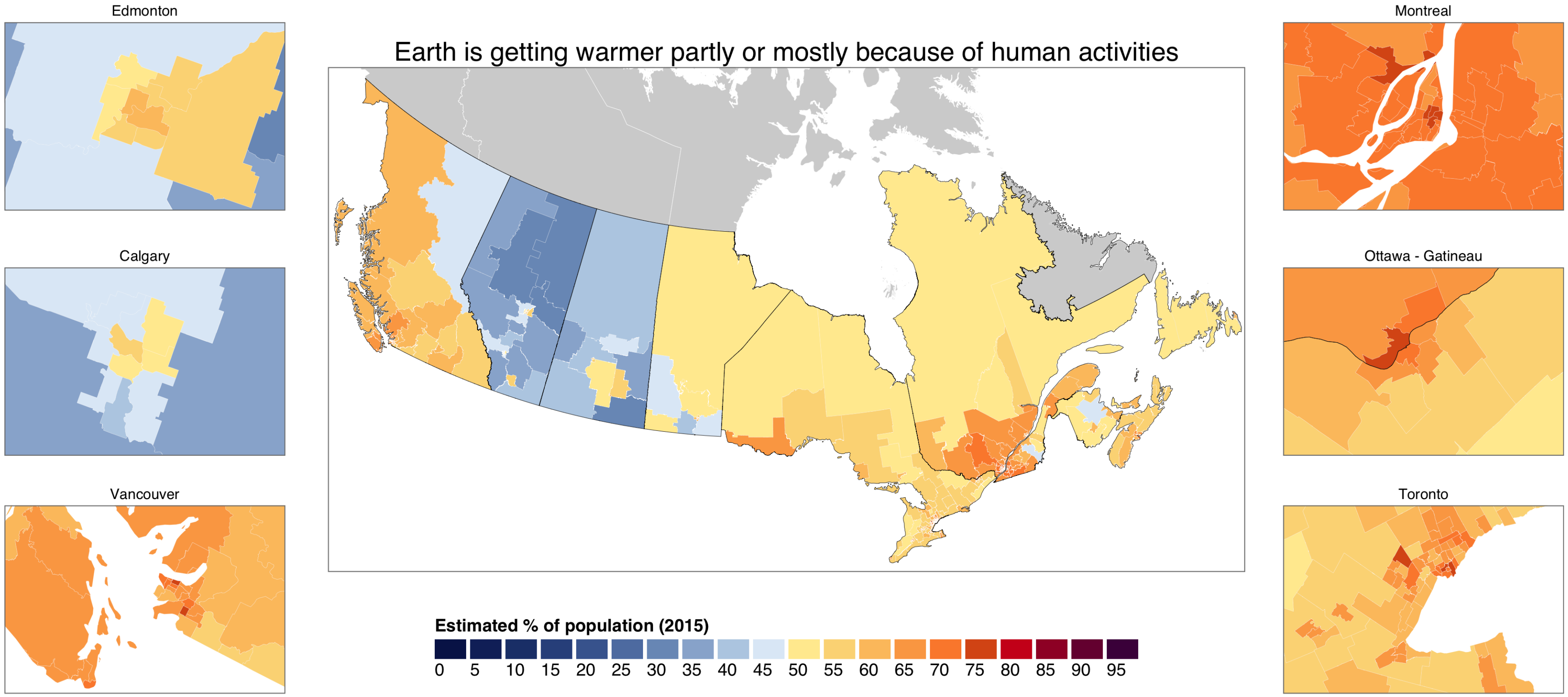

Figure 8: (**S8**) Belief that Earth is getting warmer partly or mostly because of human activities at the federal electoral district (riding) level.

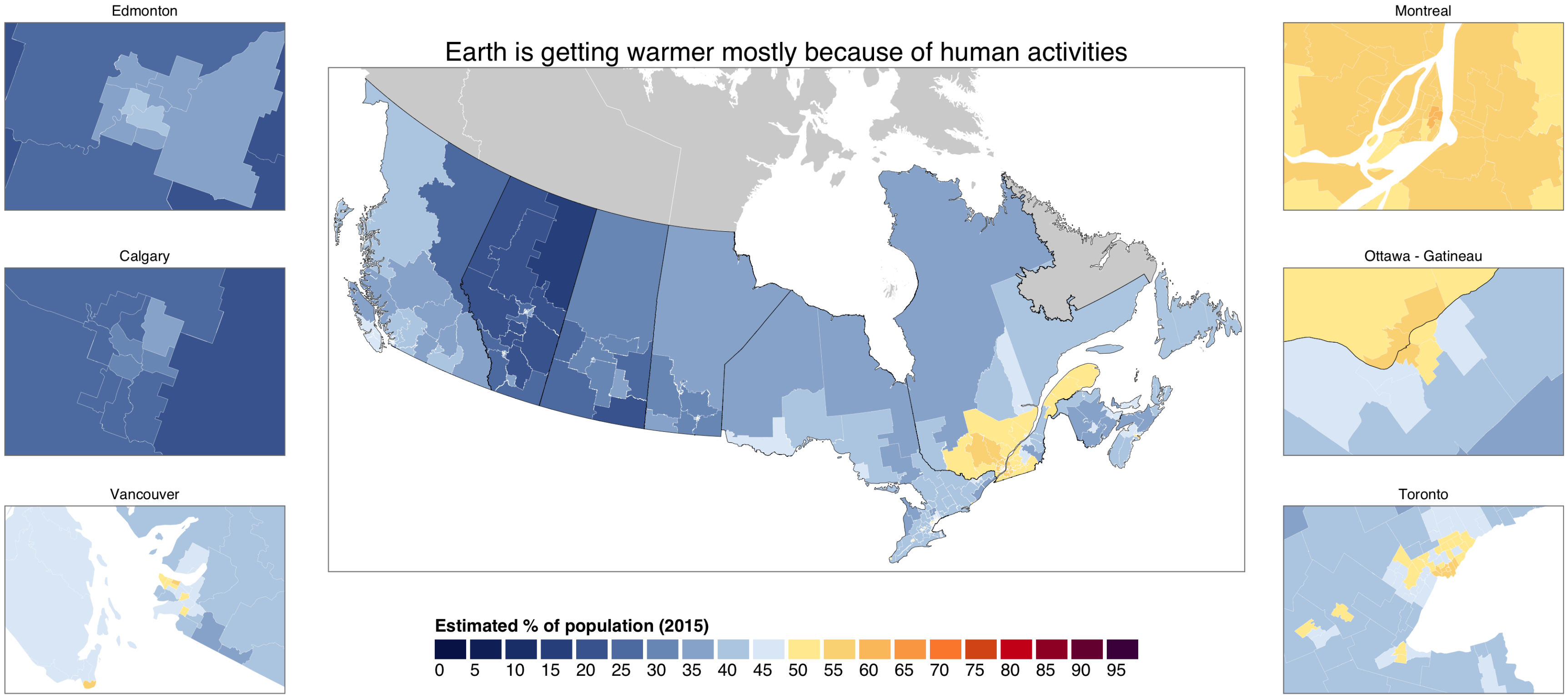

Figure 9: (**S9**) Belief that Earth is getting warmer mostly because of human activities at the federal electoral district (riding) level.

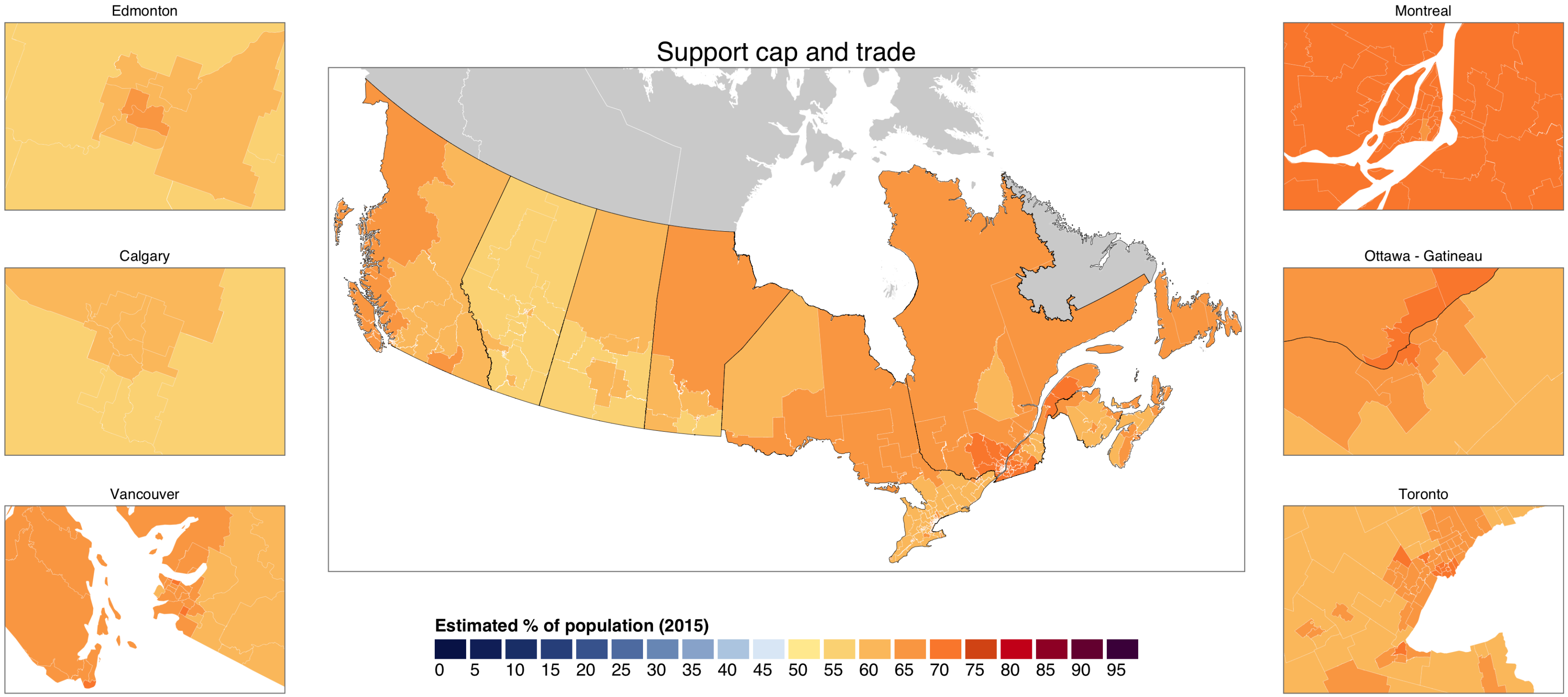

Figure 10: (**S10**) Support for cap and trade at the federal electoral district (riding) level.

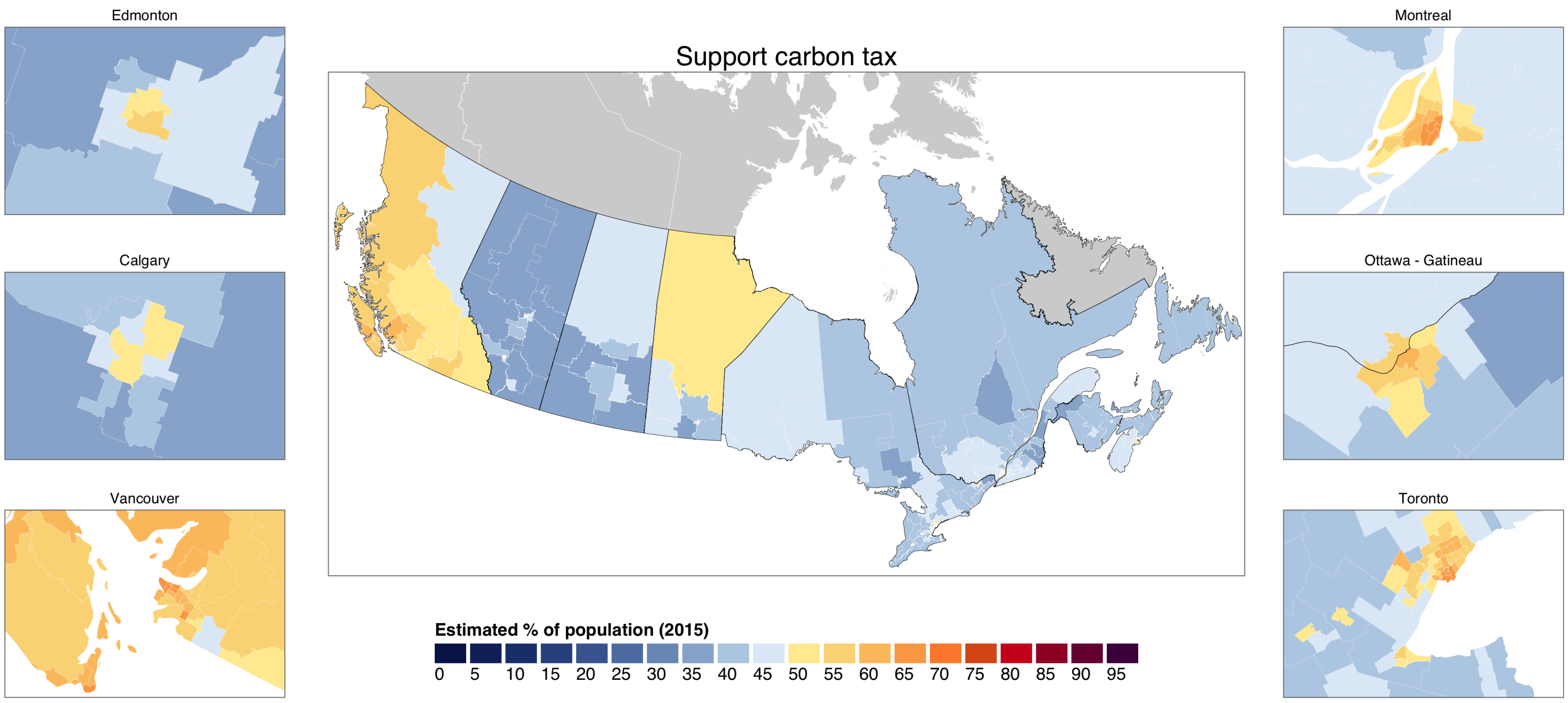

Figure 11: (**S11**) Support for carbon tax at the federal electoral district (riding) level.
